# Supplementary material for: A fault-intrusion-tolerant system and deadline-aware algorithm for scheduling scientific workflow in the cloud
Source: PeerJ Comput Sci. 2021 Nov 2;7:e747. doi: 10.7717/peerj-cs.747 (PMC8576554; doi:10.7717/peerj-cs.747)
Supplement: Supplemental Information 6 [file peerj-cs-07-747-s006.zip › Myproject1.1/docs/org/cloudbus/cloudsim/power/class-use/PowerHostUtilizationHistory.html]

Uses of Class org.cloudbus.cloudsim.power.PowerHostUtilizationHistory


JavaScript is disabled on your browser.


- Overview
- Package
- Class
- Use
- Tree
- Deprecated
- Index
- Help

- Prev
- Next

- Frames
- No Frames

- All Classes

## Uses of Class org.cloudbus.cloudsim.power.PowerHostUtilizationHistory

- Packages that use PowerHostUtilizationHistory

  | Package | Description |
  | --- | --- |
  |  |  |
  | --- | --- |
  | org.cloudbus.cloudsim.power |  |
- - ### Uses of PowerHostUtilizationHistory in org.cloudbus.cloudsim.power

    Methods in org.cloudbus.cloudsim.power that return types with arguments of type PowerHostUtilizationHistory

    | Modifier and Type | Method and Description |
    | --- | --- |
    |  |  |
    | --- | --- |
    | `protected java.util.List<PowerHostUtilizationHistory>` | PowerVmAllocationPolicyMigrationAbstract.`getOverUtilizedHosts()` Gets the over utilized hosts. |

    Methods in org.cloudbus.cloudsim.power with parameters of type PowerHostUtilizationHistory

    | Modifier and Type | Method and Description |
    | --- | --- |
    |  |  |
    | --- | --- |
    | `protected double` | PowerVmAllocationPolicyMigrationInterQuartileRange.`getHostUtilizationIqr(PowerHostUtilizationHistory host)` Gets the host utilization iqr. |
    | `protected double` | PowerVmAllocationPolicyMigrationMedianAbsoluteDeviation.`getHostUtilizationMad(PowerHostUtilizationHistory host)` Gets the host utilization mad. |

    Method parameters in org.cloudbus.cloudsim.power with type arguments of type PowerHostUtilizationHistory

    | Modifier and Type | Method and Description |
    | --- | --- |
    |  |  |
    | --- | --- |
    | `protected java.util.List<java.util.Map<java.lang.String,java.lang.Object>>` | PowerVmAllocationPolicyMigrationAbstract.`getMigrationMapFromUnderUtilizedHosts(java.util.List<PowerHostUtilizationHistory> overUtilizedHosts)` Gets the migration map from under utilized hosts. |
    | `protected java.util.List<? extends Vm>` | PowerVmAllocationPolicyMigrationAbstract.`getVmsToMigrateFromHosts(java.util.List<PowerHostUtilizationHistory> overUtilizedHosts)` Gets the vms to migrate from hosts. |
    | `protected void` | PowerVmAllocationPolicyMigrationAbstract.`printOverUtilizedHosts(java.util.List<PowerHostUtilizationHistory> overUtilizedHosts)` Prints the over utilized hosts. |

- Overview
- Package
- Class
- Use
- Tree
- Deprecated
- Index
- Help

- Prev
- Next

- Frames
- No Frames

- All Classes
